# Supplementary material for: The pharmacological perturbation of brain zinc impairs BDNF-related signaling and the cognitive performances of young mice
Source: Sci Rep. 2018 Jun 27;8:9768. doi: 10.1038/s41598-018-28083-9 (PMC6021411; doi:10.1038/s41598-018-28083-9)
Supplement: Supplementary file 1 — Supplementary figures and table [file 41598_2018_28083_MOESM1_ESM.pdf]

The pharmacological perturbation of brain zinc impairs BDNF-related signaling and the cognitive performances of young mice

Valerio Frazzini *, Alberto Granzotto *, Manuela Bomba, Noemi Massetti, Vanessa Castelli, Marco d'Aurora, Miriam Punzi, Mariangela Iorio, Alessandra Mosca, Stefano Delli Pizzi, Valentina Gatta, Annamaria Cimini, Stefano L. Sensi

Supplementary figure 1

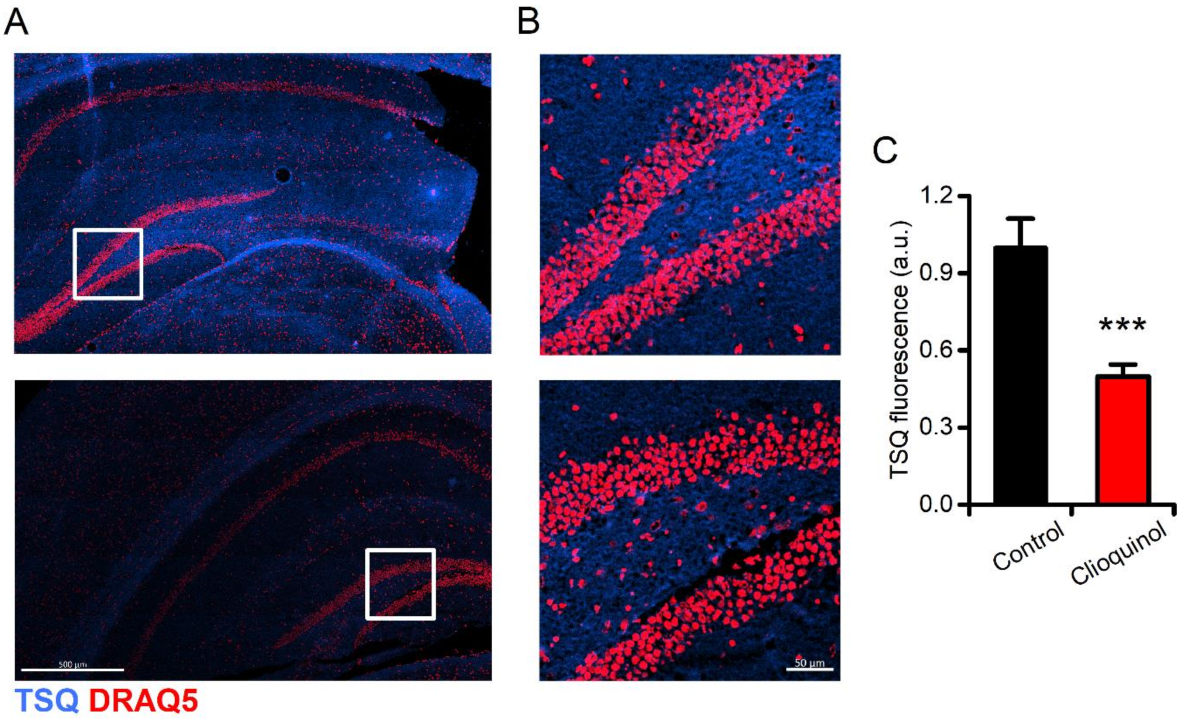

Supplementary figure 1. CQ administration reduces chelatable zinc in the brain. TSQ staining was employed to assess levels of chelatable Zn^{2+} in the hilus of the hippocampal dentate gyrus (DG). (A) Representative images of brain sections obtained from vehicle- (left) and CQ-treated (right) mice. (B) Magnification of the hilus of the DG as in (A). (B) The bar graph shows the quantification obtained from images in A (TSQ normalized fluorescence in vehicle-treated 1.00±0.11 vs 0.49±0.04 in CQ-treated mice, n=6-8 brain slices from vehicle- and CQ-treated mice, p<0.001). "***" indicates p<0.001

Supplementary figure 2

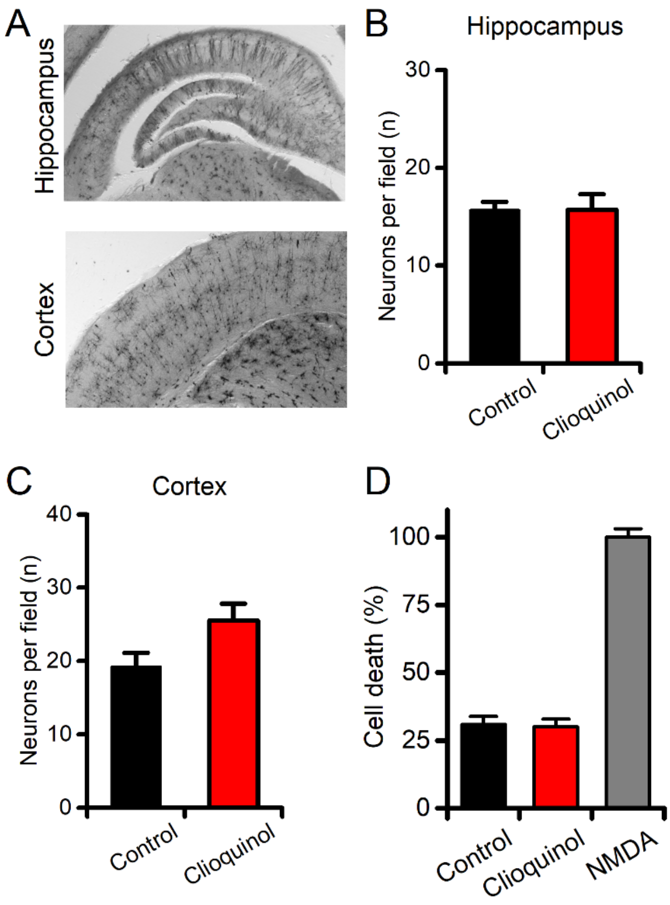

Supplementary figure 2. CQ supplementation does not affect neuronal viability. (A) Representative images of Golgi-stained brain sections obtained from vehicle- (left) and CQ-treated (right) mice. The Golgi-staining was employed as an indirect index of neuronal viability. The viability was evaluated as the average number of neurons per field in slices obtained from vehicle- and CQ-treated mice. (B-C) Bar graphs depict the quantification of the average number of Golgi-stained neurons per field (Hippocampus: 15.62±0.87 for control vs 15.72±1.55 for CQ-treated mice, n=4 per condition, p=0.87; Cortex: 19.20±1.92 for control vs 25.52±2.30 for CQ-treated mice, n=4 per condition, p=0.08). (D) Bar graph depicts the viability of cultured hippocampal neurons exposed to CQ (10 µM) or vehicle for 3 days. Neuronal viability was assessed by LDH efflux assay and results normalized to a set of experiments in which almost complete neuronal death was achieved by exposing cells to toxic levels of NMDA (300 µM + 10 µM glycine) for 24h (Control 30.94±2.88 vs 30.08±2.81 in CQ-treated cultures, n=9 per condition, p=0.83).

Supplementary table 1. Statistics for the WB experiments shown in main text.

		Control	Clioquinol			
		Mean±SEM	Mean±SEM	p	n	Significance
**BDNF**	Hippocampus	1.40±0.07	0.91±0.06	0.002	4	**
	Cerebellum	0.21±0.04	0.29±0.07	0.34	4	
	Cortex	1.81±0.06	1.30±0.14	0.015	4	*
	Striatum	0.86±0.09	0.50±0.06	0.022	4	*
**TrkB**	Hippocampus	1.13±0.05	0.66±0.12	0.013	4	*
	Cerebellum	0.54±0.06	0.49±0.06	0.57	4	
	Cortex	2.00±0.08	1.71±0.07	0.03	4	*
	Striatum	1.32±0.07	0.78±0.04	0.001	4	**
**pTrkB/TrkB**	Hippocampus	0.74±0.09	0.84±0.21	0.66	3	
	Cerebellum	0.38±0.04	0.54±0.19	0.47	3	
	Cortex	0.56±0.07	0.43±0.01	0.15	3	
	Striatum	0.49±0.01	0.55±0.09	0.56	3	
**PSD95**	Hippocampus	0.89±0.03	0.69±0.04	0.02	3	*
	Cerebellum	0.24±0.03	0.20±0.03	0.45	3	
	Cortex	1.62±0.04	1.29±0.04	0.007	3	**
	Striatum	1.20±0.05	0.71±0.08	0.008	3	**
**pERK5/ERK5**	Hippocampus	0.91±0.08	0.34±0.03	0.002	3	**
	Cerebellum	0.41±0.03	0.43±0.01	0.57	3	
	Cortex	0.80±0.03	0.52±0.06	0.019	3	*
	Striatum	0.54±0.08	0.26±0.02	0.03	3	*
**proBDNF**	Hippocampus	1.22±0.06	0.91±0.05	0.02	3	*
	Cerebellum	0.28±0.04	0.20±0.02	0.18	3	
	Cortex	1.19±0.10	0.64±0.07	0.01	3	*
	Striatum	0.63±0.05	0.23±0.05	0.006	3	**
**p75NTR**	Hippocampus	1.16±0.07	1.49±0.04	0.01	3	*
	Cerebellum	0.30±0.03	0.44±0.06	0.11	3	
	Cortex	2.47±0.03	2.74±0.02	0.002	3	**
	Striatum	0.22±0.03	0.93±0.03	<0.001	3	***
**$pERK_{1,2}$/ $ERK_{1,2}$**	Hippocampus	0.54±0.05	0.88±0.08	0.03	3	*
	Cerebellum	0.20±0.01	0.24±0.01	0.10	3	
	Cortex	1.59±0.13	2.13±0.07	0.02	3	**
	Striatum	0.98±0.01	1.22±0.069	0.03	3	**

Data are represented as mean ± SEM. "*" indicates $p < 0.05$, "**" indicates $p < 0.01$, and "***" indicates $p < 0.001$.
